# RNA 3-dimensional structural motifs as a critical constraint of viroid RNA evolution

**DOI:** 10.1371/journal.ppat.1006801

**Published:** 2018-02-22

**Authors:** Ying Wang, Craig L. Zirbel, Neocles B. Leontis, Biao Ding

**Affiliations:** 1 Department of Biological Sciences, Mississippi State University, Starkville, Mississippi, United States of America; 2 Department of Mathematics and Statistics, Bowling Green State University, Bowling Green, Ohio, United States of America; 3 Department of Chemistry and Center for Biomolecular Sciences, Bowling Green State University, Bowling Green, Ohio, United States of America; 4 Department of Molecular Genetics, The Ohio State University, Columbus, Ohio, United States of America; 5 The Center for RNA Biology, The Ohio State University, Columbus, Ohio, United States of America; University of Kentucky, UNITED STATES

## Introduction

Viroids are circular noncoding RNAs infecting plants [1, 2]. During infection, viroids, like RNA viruses, generate swarms of sequence variants called quasispecies [3, 4]. Viroids in Avsunviroidae family replicate in chloroplasts and display the highest mutation rates among all living entities [5]. Viroids in Pospiviroidae family replicate in the nucleus with a relatively lower mutation rate resembling some RNA viruses [6]. Those sequence variants generated during replication are described by the concept of sequence space, which harnesses a geometric representation to illustrate genetic similarities via physical distances. Given the high mutation rate and fast propagation, viroid RNAs have a potentially large sequence space for the evolution of new variants. However, in reality, they use only a small fraction of this space. Constraints of viral sequence space may include genome size, replication fidelity, error thresholds, host or tissue tropism, etc. These factors have been nicely reviewed elsewhere [3, 7, 8] and are not the focus of this Pearl. In addition, RNA secondary structures have been considered, though not adequately, as a constraint factor [8]. Viroids, in contrast to viruses, entirely rely on their RNA structural motifs for function due to their noncoding nature, which offers insights into their capacity to explore regions of sequence space influenced by RNA structures.

Here, we describe that 3-dimensional (3D) structural motifs formed by non–Watson-Crick (non-WC) base pairs in viroid RNAs act as a critical constraint for the sequence space of viroid genome evolution. This constraint operates because RNA 3D motifs can play crucial roles by mediating (1) RNA–RNA interactions for the folding of a part or a whole of RNA into a distinct tertiary conformation and (2) RNA–protein interactions. Therefore, mutations in a 3D motif that do not disrupt the structure and function will be retained in the population, whereas mutations that disrupt the 3D structures of motifs, and consequently the function, will be lost.

## Question 1: What are the features of local RNA 3D structural motifs?

**Fig 1:**
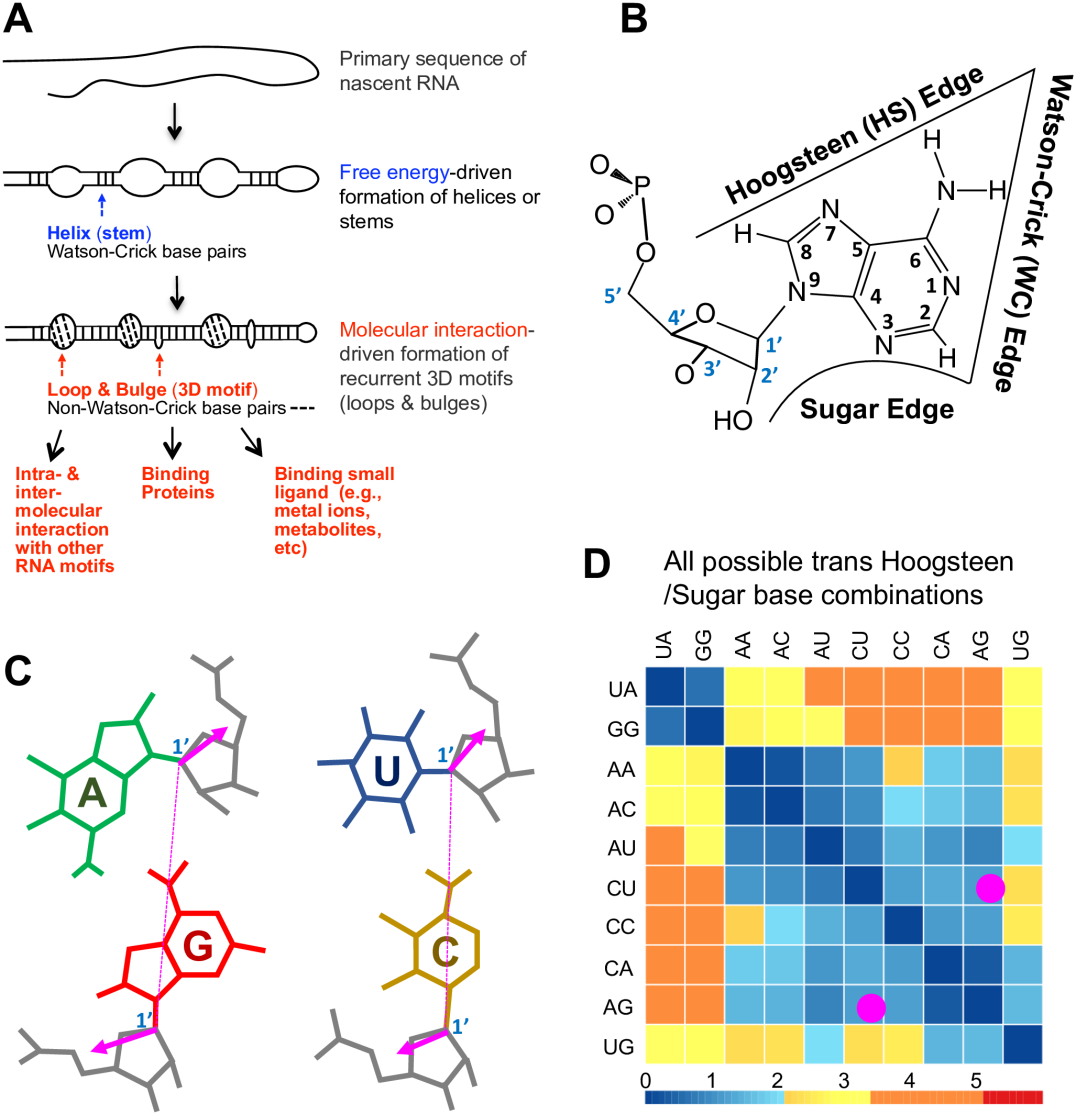
RNA structure basis. (A) Simple illustration of RNA primary, 2D, and 3D structures. (B) Three edges of adenine nucleotides. (C) Isosteric AG tHS and CU tHS base pairs. Glycosidic bond orientations are highlighted with magenta arrows. C1’-C1’ distances are highlighted with dashed magenta lines. (D) tHS IsoDiscrepancy Index heat map from the RNA Basepair Catalog. Any base combinations in the tHS family are listed, and AG vs CU is marked in magenta dot. Lower numeric value (less than 2.2 in blue color) dictates isosteric base pairs. Values between 2.2 and 3.5, colored in yellow, show nearly isosteric base pairs. Values above 3.5, colored in orange or red, dictate the nonisosteric base pairs. A, adenine nucleotides; C, cytocine nucleotides; G, guanine nucleotides; tHS, Trans Hoogsteen/Sugar edge; U, uridine nucleotides.

RNA 3D structures, to a first approximation, are composed of helices (formed by contiguous WC base pairs such as adenine [A]–uridine [U], guanine [G]–cytosine [C], and GU base pairs) and loops, both of which are shown in RNA 2D structures (Fig 1A). The loops are usually structured by additional interactions, including non-WC base pairs, base–backbone interactions, and base stacking (Fig 1A). In larger RNAs, these “local” loops can bind to helices or other loops distant in the 2D structure, stabilizing a larger-scale 3D structure. The loops have been described in detail by atomic-resolution crystallography and NMR spectroscopy studies [9]. Loop geometries and interaction details are typically conserved in homologous positions across species. Those RNA loop geometries that recur in nonhomologous positions of unrelated RNA molecules, with at most minor variations, are referred to as recurrent RNA 3D motifs [10, 11].

## Question 2: What are non-WC base pairs?

Each RNA base has 3 edges, the WC, Hoogsteen, and Sugar edges, that can potentially hydrogen bond (H-bond) with other base edges in loop motifs (Fig 1B) [12]. According to the relative positions of glycosidic bonds, for each pair of interacting edges, there are 2 possible orientations, called “cis” (together) and “trans” (opposed). In total, there are 12 base-pairing geometries. Sequence variations observed for paired positions in RNA motifs are typically isosteric, meaning that base substitutions occupying similar space are potentially interchangeable without disrupting 3D structures [13]. To qualify, those base interactions should (1) use the same edges for interaction, (2) share the same orientations (cis or trans) of glycosidic bonds, and (3) occupy the same C1’-C1’ distance in space. Base pair isostericity reduces the range of base substitution in 3D motifs (Fig 1C). Features of all possible RNA base pairings, including edge interactions, glycosidic bond orientations, and C1’-C1’ distances, are displayed in the RNA Basepair Catalog (http://ndbserver.rutgers.edu/ndbmodule/services/BPCatalog/bpCatalog.html). The Catalog provides a numerical measure of the degree of isostericity among different base combinations for each of the 12 base-pairing geometries, displayed in interactive heat maps, illustrated by AG versus CU trans Hoogsteen/Sugar Edge base pairs in Fig 1D.

## Question 3: Why are 3D structures of RNA loop motifs critical for function?

In a regular RNA helix, only the minor groove is easily accessible to proteins, while the major groove is too narrow for inserting alpha helixes, as occurs in DNA–protein complexes. The minor groove (sugar) edges of the nucleotides display a smaller difference between AU and GC base pairs than the major groove, but some amino groups, such as GN2 in guanine nucleotide, can sometimes constrain RNA sequence variations when H-bonding with proteins. More common functional sites are the loop regions of an RNA that provide specific binding locations for proteins or other molecules. Non-WC base pairs in RNA loops expose WC edges and widen the major groove. The WC edges are more distinct across the 4 bases, which allows for specific interactions critical for function.

## Question 4: What is the evidence that RNA 3D motifs are critical for viroid infection?

**Fig 2:**
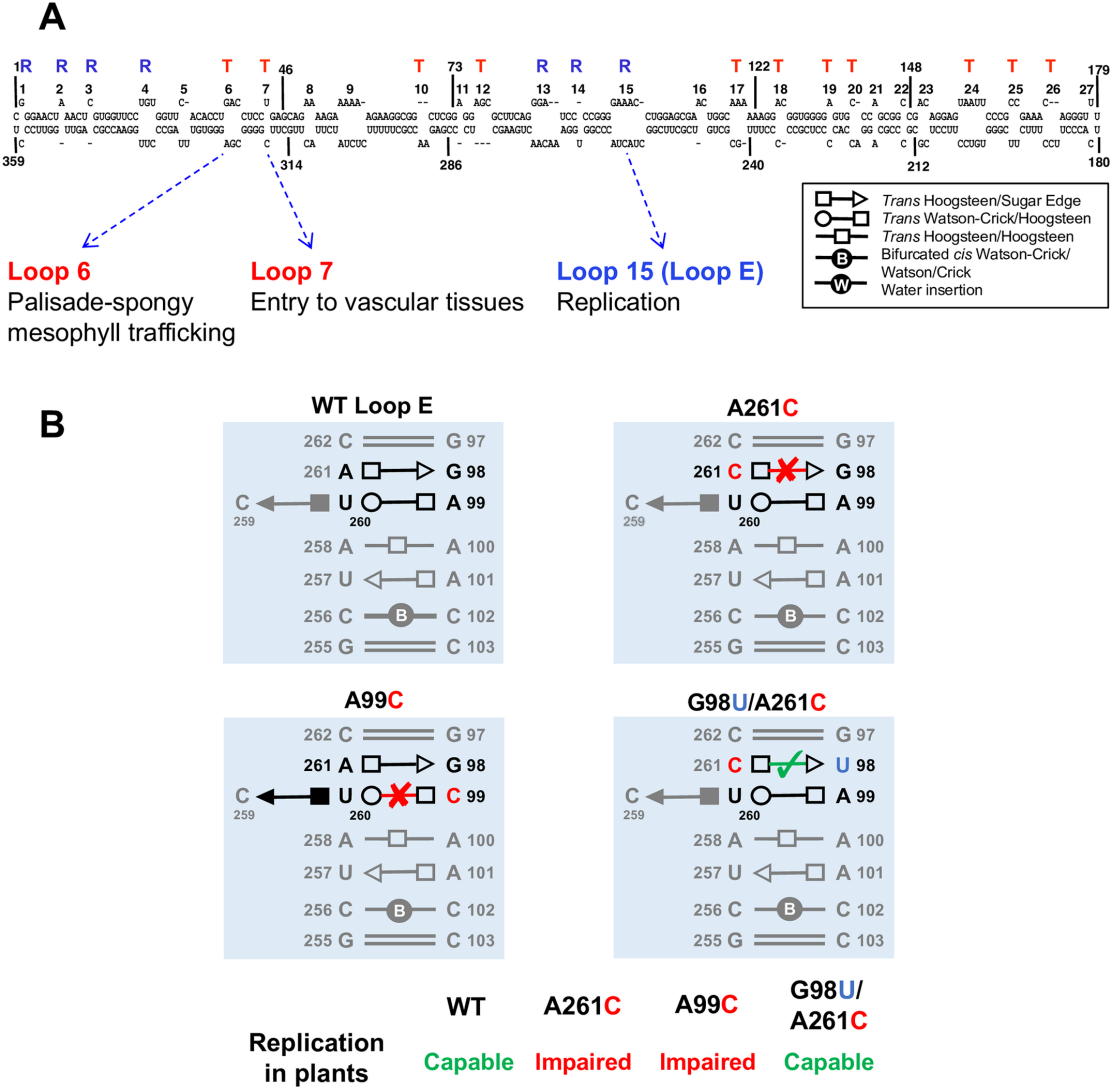
PSTVd RNA structures. (A) The 2D organization of PSTVd RNA genome. 3D structural arrangements and the function of loop 6, loop 7, and loop E are listed [17–19]. “T” and “R” depict the functions in “trafficking” and “replication,” respectively [16]. (B) Disruptive and compensatory PSTVd loop E mutants predicted by isostericity [17]. Illustration for the replication of PSTVd variants in tomato plants, verified by northern blots [17], is shown in the lower panel. PSTVd, potato spindle tuber viroid; WT, wild-type.

RNA secondary structures of potato spindle tuber viroid (PSTVd), the type species of Pospiviroidae family, have been well characterized through chemical mapping (Fig 2A) [14, 15]. Noteworthy is that both studies, including the recently developed Selective 2’-hydroxyl acylation analyzed by primer extension experiments, support the existence of base pairs within loop motifs [14, 15], and 18 out of 27 RNA loops in the PSTVd genome are critical for either replication or systemic spreading [16], both of which are commonly used for assessing the fitness of viruses as surrogates [3].

Three-dimensional non-WC base pair arrangements in several PSTVd RNA motifs were annotated recently. Zhong et al. [17] analyzed the PSTVd loop E motif and validated the 3D structural arrangements therein. Interestingly, variants predicted to form nonisosteric base pairs (A99C and A261C) impair the replication capacity, while compensatory mutants (G98U/A261C) predicted to recover the original non-WC base pair restore the replication capacity (Fig 2B), demonstrating that isostericity dictates the function of RNA motifs.

Following this study, 3D structural arrangements of 2 additional PSTVd motifs have been shown to play critical roles [18, 19]. U43/C318 forms a single base pair motif (cis WC/WC) with a water insertion, termed loop 7, that regulates the entry of PSTVd to vascular tissues for spreading [19]. In addition, the neighboring loop 6 governs trafficking from palisade mesophyll to sponge mesophyll in plant leaves by forming specific non-WC base pairs [18]. Noteworthy is that saturated mutational analyses showed that the functional variants in each loop share isosteric structures.

## Question 5: What is the evidence for RNA 3D structural motifs constraining viroid evolution?

Because some RNA 3D motifs control viral infection, strong selective pressures exist for maintaining the 3D motif structures that constrain the variation in sequence space. Mutational analyses on loop E, loop 6, and loop 7 all support this [17–19]. Taking loop 6 as an example, the 3D structure of this 3 × 3 loop was predicted using sequence-based homology search against RNA structure database [20], and the predicted model was consistently supported by data from functional mutagenesis analyses and chemical probing [19]. PSTVd loop 6 has a total of 4^6^ possible sequence combinations, but there are only 8 functional variants out of 49 possible isosteric combinations [19]. Therefore, isostericity in RNA 3D motifs significantly reduced the sequence variations in PSTVd loop 6 by 84-fold (= 4^6^/49) and testing for function by an additional factor of 6 (= 49/8), indicating that RNA 3D structural motifs serve as a critical constraining factor.

## Question 6: How do viroids adapt to new environments while under constraints to form RNA 3D motifs?

While maintaining the 3D structure of RNA loop motifs is pivotal, isosteric base substitutions may allow infection of new tissues or hosts. Previously, no infectious PSTVd strain for *Nicotiana tabacum* (tobacco) was observed in nature. However, in planta selection assays identified the C259U substitution in PSTVd loop E that led to the emergence of a new infectious strain for tobacco [21]. A subsequent study in transgenic tobacco also showed substitutions in loop E (C259U or U257A) enabling PSTVd infection of tobacco [22]. Both substitutions are predicted to be isosteric with the original wild-type (WT) sequences [17]. Therefore, isosteric base substitutions in loop E can both maintain the local 3D structure and allow for the emergence of new infectious PSTVd variants.

## Conclusions and perspectives

Maintaining structures of RNA 3D motifs serves as a critical constraint of viroid evolution. In RNA 3D motifs, isosteric base substitutions in noncanonical base pairs are required to maintain 3D motif structure, greatly reducing the range of possible base substitutions. Maintaining functional interactions with proteins reliant on specific nucleotide–residue combinations further reduces the space of possible base changes.

RNA 3D motifs may be a constraint for viruses as well. Despite differences in their genetic makeups and unique infection and evolution pathways, different viral and viroid RNAs should all share one common property: RNA 3D motif–based RNA–RNA, RNA–protein, and RNA–small ligand interactions necessary for completing life cycles [23–27]. Therefore, understanding how RNA 3D structural motifs play a role in viral infection and their exploration for regions of sequence space may potentially improve the prediction of outbreaks of new viruses.
